# Data covering soil management practices and farm characteristics on Swiss arable farms

**DOI:** 10.1038/s41597-025-05731-0

**Published:** 2025-08-23

**Authors:** Charles Rees, Lorin Ineichen, Robert Finger, Christian Grovermann

**Affiliations:** 1 Department of Food System Sciences, Research Institute for Organic Agriculture (FiBL), Frick, Switzerland; 2 Agricultural Economics and Policy Group, ETH Zürich, Zürich, Switzerland

**Keywords:** Agriculture, Sustainability

## Abstract

Soil health is the cornerstone of sustainable agriculture, but studies have shown that agricultural soils are degrading due to inadequate soil management practice adoption. To understand the *status quo*, we surveyed 2,728 Swiss arable farms in 2024. The dataset captures the soil management practices used alongside the decision-making surrounding their implementation in the 2022/2023 production season. Four core components are covered: (1) farm and farmer characteristics, including gender, age, experience, labour, farm size and Agri-environmental scheme participation; (2) detailed records of twelve arable soil management practices, including uptake extent, number of years used, perceived knowledge and peer adoption; (3) farmers’ priorities for soil health and their assessment of key agricultural challenges; and (4) production data for a subset of farms cultivating milling wheat, including wheat area, wheat yield and input application rates. We enriched the dataset with linked secondary plot-level census data. This combined dataset provides a comprehensive resource that enables the analysis of current farming practices, knowledge gaps and challenges to maintain and improve the health of arable soils.

## Background & Summary

In this data descriptor we present data from 2,728 Swiss arable farms regarding the use of soil management practices for improving soil health on their arable land during the 2022/2023 production season^1^. Between December 2023 and January 2024, we collected a comprehensive dataset via an online survey from arable farms across Switzerland. The survey captured a breadth of information on farmers’ knowledge, preferences, and practices related to soil health. Additionally, this data descriptor incorporates two supplementary datasets: (1) wheat production data and (2) a secondary dataset extracted from freely available national geographic and farm census data, which further enriches the understanding of the agricultural landscape and complements the survey data^1^. The supplementary datasets can be linked to the main dataset through a common key variable.

Before presenting the data collection protocol and the detailed variables of the datasets, we first provide a brief introduction to soil health in European agriculture and then describe the study area and associated policy background in Switzerland at the time of our study.

## Soil Health and Agriculture

Soil is an indispensable resource that is vital for the continued functioning of ecosystems as well as for ensuring the long-term supply of food^2–4^. Maintaining and improving the health of agricultural soils is critical to achieve the sustainable transformation goals set for the food system^5^. In addition to food production, agricultural soils provide many additional essential ecosystem services that are crucial to support life on earth^2,4,6^. Such services include carbon sequestration and storage functions^7,8^, the regulation of water quality and quantity flowing into water courses^2,9^ and provision of habitat for a large array of soil fauna and flora^10–12^.

Soil health is increasingly under threat from intensive agricultural practices and changing weather patterns^13^. This includes higher rainfall intensities, which are projected to accelerate soil erosion and result in substantial economic losses in the coming decades^14,15^. Consequently, improving and maintaining agricultural soil health is high on the agenda of policymakers in Switzerland, in Europe and around the globe^16–18^.

In Europe, arable farmers are encouraged to adopt beneficial soil management strategies: some of which are incentivised through Agri-environmental schemes^19^. Practices such as reduced tillage, organic matter addition, crop rotation and maintaining permanent soil cover are often subsidised and have been shown through field trials to improve soil health^20^, enhance resilience to environmental stressors^21^, and support the provision of soil ecosystem services^22–24^. Yet, to steer the transition towards more sustainable and healthy agricultural soils in an effective and efficient manner, it is imperative to understand the current practices adopted on farms, their impacts and the potential drivers or barriers to further implementation^25,26^ within their geographic and cultural context^27,28^. However, there is currently a significant gap in large-scale data on actual soil health decisions across different farming practices. The lack of such comprehensive data limits our ability to fully assess the effectiveness of current practices, understand farmers’ decision-making processes, and identify the key factors influencing the adoption of sustainable soil management. The dataset we present here fills an important gap by providing insights into the actual soil management practices of Swiss arable farmers, enabling a deeper understanding of the barriers and drivers that can shape future soil health initiatives.

## Study Area

### Swiss policy background

Switzerland launched its own national soil strategy in 2020 with eight stated targets for agriculture, including the reduction of soil compaction, prevention of soil degradation from erosion, minimising soil organic matter breakdown and avoiding soil biodiversity loss^29^. Swiss agriculture operates within a distinct policy framework, whereby farms are supported financially to a high degree by the Federal Government directly via direct subsidies (known as direct payments) as well as indirectly via border regulations targeting the import and export of agricultural products^30–32^.

**Fig. 1:**
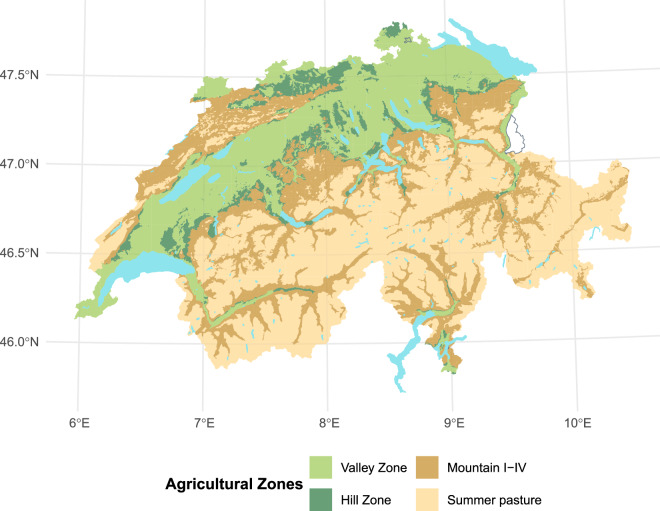
The spatial distribution of agricultural zones in Switzerland. Arable farms are mainly situated in valley and hill zones. The classification is based on data provided by the Federal Office for Agriculture of Switzerland.

As a prerequisite to receiving direct payments, farmers in Switzerland must provide proof of ecological performance (German name Ökologischer Leistungsnachweis), which sets out basic environmental and land management requirements. Once Swiss farmers fulfil the proof of ecological performance, which is the case for 98% of farms^33^, they become eligible for direct payments classified within five different pillars. It is important to note that Swiss area-based agricultural support payments are also graduated across distinct agricultural zones (Fig. 1), this is due to the diverging landscapes prevalent across Switzerland. Two of these area-based payments are the “cultural landscape” and “food security” contributions. However, farmers may also enrol voluntarily for further, mainly action-based, schemes known as “production system”, “landscape quality” and “biodiversity” contributions. These require that farmers undertake additional practices and meet additional criteria.

The Swiss Federal Office of Agriculture has two subsidy schemes within the production system contribution pillar, introduced in 2023, directly targeting soil. These include the reduced tillage scheme (CHF 250/ha enrolled) and the continuous soil cover scheme (CHF 200/ha enrolled)^34^. Additionally, several core concepts of sustainable soil management are a statutory minimum requirement for receiving direct payments, such as appropriately diversified crop rotations^35^. However, many beneficial soil management practices were not directly subsidised in Switzerland at the time of our survey.

### Swiss agricultural sector

As of 2023, the Swiss farming sector comprised 47,719 farm enterprises, with each covering an average farm size of 21.84 hectares, operating under one of three production systems: conventional, integrated or organic production^36,37^. Conventional farming meets basic legal standards, whilst integrated production is a state-recognised system that emphasises resource efficiency, soil protection, and reduced – but not eliminated – use of synthetic pesticides and fertilisers. Within the integrated production framework, various programs exist that define different levels of input use restrictions^38^. Organic farming on the other hand follows stricter ecological and animal welfare standards and prohibits the use of synthetic pesticides and mineral fertilisers altogether^39^.

Of the total number of farms across all production systems, only 19,227 are involved in any form of grain production (40.40% of farms) and the total area of arable fields in Switzerland is 274,896.24 hectares (26.38% of all farmed land), as of 2023. Winter wheat is the most widely produced arable crop, covering 78,075.96 hectares (28.40% of the arable area) and amounting to a total inland production of 413,805 tonnes in 2023 (80.63% going to human consumption)^40–42^. A total of 148,880 people are employed in agriculture, 44.21% on a permanent basis, and 71.03% of farms are full-time enterprises based on figures from 2023^36^.

**Fig. 2:**
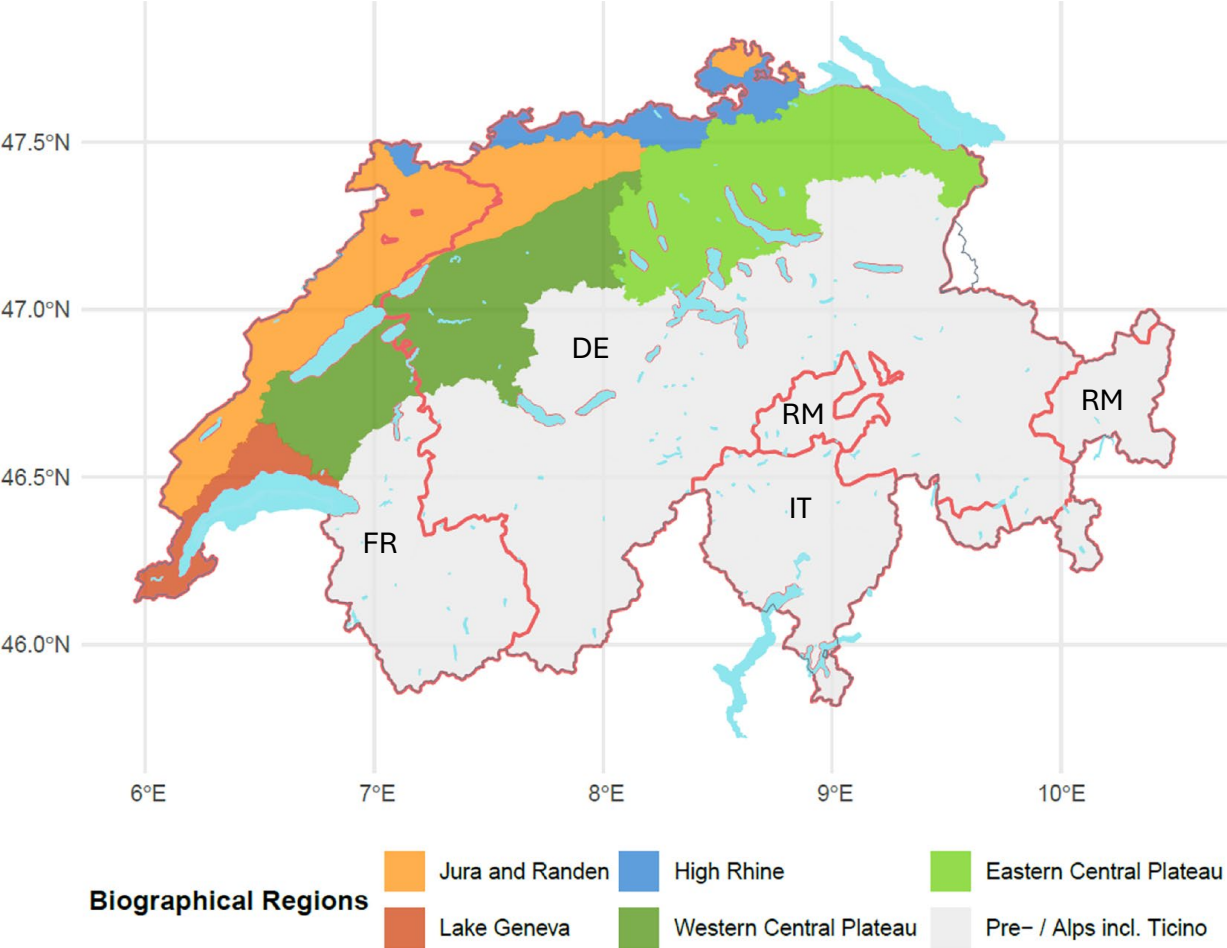
The biogeographical landscape zones of Switzerland with the main linguistic borders listed - German (DE), French (FR), Italian (IT) and Romansh (RM). The map is based on data from the Federal Office for the Environment and the Federal Statistical Office of Switzerland.

Geographically, Switzerland can be divided into several different landscape regions: the Jura mountains to the north-west, the Swiss plateau in the centre, and the Alps in the south and east. Arable farming in Switzerland is primarily concentrated in the Swiss Plateau^42^. The predominant climate is highly dependent on the topography but in the Swiss plateau, there is a continental climate which is typified by cold winters and warm summers (for further information on the climate of Switzerland, see: The climate of Switzerland - MeteoSwiss). Figures 1 and 2 provide an overview of the regional diversity within Swiss agriculture: Fig. 1 above illustrates the country’s agricultural zones, while Fig. 2 below depicts its main biogeographical regions. Each biographical region is shaped by its unique geographic, climatic, and ecological conditions, as well as the agricultural practices and land use strategies adopted by farmers. In addition to these physical characteristics, Switzerland is also shaped by four distinct cultural-linguistic regions—German, French, Italian, and Romansh. The borders of these language regions are indicated in Fig. 2.

## Methods

### Sampling procedure

In the context of the Horizon Europe Project “InBestSoil”, the data collection focused on arable management practices in Switzerland. Specifically, those practices related to soil health and soil conservation undertaken within the 2022/2023 production season. Farm selection for the survey was based on specific criteria to ensure that the data collection accurately represented arable agricultural practices in Switzerland. These criteria were designed to target farms that were significantly involved in arable agriculture, which is crucial for assessing arable soil health management practices. Eligible farms were required to meet the following criteria:

- Grow wheat in the preceding season (2021/2022).
- Farm at least 3 hectares of arable land in the preceding season (2021/2022).
- Arable land must have comprised at least 20% of the total farmed area in the preceding season (2021/2022).

We entered a data sharing agreement with the Federal Office of Agriculture to enable our survey campaign via access to contact information of all farmers who met the above selection criterion (see the supplementary material in the data repository for a copy of this contract)^1^. The Federal Office of Agriculture implemented our selection criterion on the agricultural data that they collect on a yearly basis from the direct payment applications of all Swiss farmers. Note, at the time of our application to the Federal Office of Agriculture, data for the production season 2022/2023 was not available. This is why we use data from the preceding production season for specifying the selection criteria, as this was the latest data available at the time, from which the Federal Office of Agriculture could make an assessment of which farm contact details to share with us for the survey.

In August 2023, we received the contact details of 15,023 farmers who qualified for the survey from the Federal Office of Agriculture’s records. The information we received included the email address, farm identification number, language spoken, name and form of address. However, as per our data sharing agreement with the Federal Office of Agriculture, this data was allowed exclusively for our use in this project and cannot be shared with any outside partner not party to the aforementioned data sharing contract. The contact data of farmers that was received from the Federal Office of Agriculture will be kept for the duration of the InBestSoil project and stored securely on private institutional servers in encrypted files. All contact information will be deleted at the conclusion of the project (December 2026) and all data presented herewith is strictly anonymised to protect the data and identities of the farmers who took part in the survey. Moreover, we have taken measures to prevent any farmers from being identified via their answers (for example variables such as manager age, wheat areas grown, location etc. have been classified into more homogenous categorical groups), which means that the data we present here is slightly different to the data that we have available for our own analyses, as agreed under the data sharing agreement with the Federal Office of Agriculture.

### Survey design and content

While adoption of agricultural practices certainly varies with farm characteristics such as size, labour availability, or participation in agri-environmental schemes, these factors alone are not sufficient to explain farmer behaviour. There is no single set of drivers that consistently predicts adoption across studies or regions^43^. Instead, adoption depends strongly on local contexts, and the interplay of economic, social, and psychological factors^44^. To capture the complexity of adoption behaviour, the survey included questions on farmers’ priorities, perceptions, self-assessed competencies, and personal goals, as well as their exposure to peer practices, participation in training and advisory services, and sources of information. These dimensions are important because farmers do not make decisions in isolation; their attitudes towards risk, innovation and environmental values can influence their decisions alongside financial considerations. Such data contribute to a more thorough understanding of the multifaceted factors influencing soil health-related decisions. The inclusion of these variables also offer valuable insights into the barriers and drivers of sustainable soil management, essential for shaping targeted and effective agricultural policies and support programs.

The full survey is available within the data repository in French, German and English^1^. The final survey was developed over the course of a year, including revisions resulting from three rounds of consultation with external stakeholders, internal consultation and testing with farmers. All participants in the survey were asked to give their informed consent by ticking a box in the online questionnaire, confirming their agreement to participate in the study. Additionally, participants consented to the linking of secondary geographical data with their responses, which was also confirmed by ticking a separate checkbox in the survey. Once the participants had agreed to these, the survey was administered uniformly following the structure outlined below. All questions appeared in the same order and, only if certain exclusion criteria were met – such as when their previous answer ruled out any further sub-questions - were some sub-questions hidden from the view of participants. Inclusive of all sub-questions, the survey contained 57 questions, and answering the questionnaire took farmers a median time of 23 minutes.

The survey design was based on previously implemented surveys regarding agricultural production practices in Switzerland^45–49^. Specifically, questions on farm information and participation in soil-related programmes were included to assess farmers’ engagement with policy incentives and voluntary schemes. The inclusion of personal characteristics aimed to understand demographic drivers of management behaviour. The questions on management practices were developed in close collaboration with experts from the soil science and agricultural extension fields, and were cross-checked with relevant literature. Data on milling wheat production and related input use were collected to link agronomic decisions with productivity outcomes. Information on structural farm characteristics, such as farm type, location, and land tenure, provides context for understanding the decision-making environment and potential constraints faced by farmers. Finally, a strong focus was placed on behavioural and attitudinal factors, including information sourcing, perceived risks, and personal goals, to account for the cognitive and motivational dimensions of farmer behaviour. The following section provides an overview of the variables investigated within each of these question groups. The collected data are documented in the accompanying datasets^1^. Each question group corresponds to a clearly defined set of columns.

#### Demographic details (Primary dataset columns B-H)

Age, duration farm responsibility, gender, full time equivalent and whether the farm succession is already secured.

#### Participation in soil health programmes (Primary dataset columns H-S)

Organic farming support, soil cover scheme, reduced tillage scheme, herbicide-free farming scheme, pesticide-free farming scheme, efficient fertiliser use, wider row planting, beneficial insect strip, precision application, cantonal soil health support, cantonal input reduction support, cantonal investment and equipment support.

#### Management Practices (Primary dataset columns T-CA)

An overview of all management practices addressed in the survey, including their descriptions and the typical machinery used, is provided in Table 1. Farmers were asked about their knowledge about the practices, the application as well as the frequency of application within the last 10 years and whether they know other farmers that use the practice. The practices covered by our survey were selected based on the input of soil scientists and agricultural extension workers based in Switzerland.

**Table 1:** Overview of management practices included in the survey through which the presented dataset was collected, with descriptions and typical machinery used for each practice listed.

Management practice	Description	Typical machinery used
*Mulch Tillage*	A non-inversion tillage method involving shallow (5–10 cm depth) cultivations. The whole soil surface is cultivated, and crop residues are incorporated into the soil surface^53,54^	Wide variety of equipment used: ~ Tined cultivator or disc harrow ~ Shallow/chisel plough ~ Rotary harrow
*Strip Tillage*	A non-inversion tillage method that involves tilling only narrow strips where crops will be planted, leaving the rest of the soil undisturbed^53,54^.	Specialised seed drill/strip planter
*Zero Tillage*	A non-inversion crop establishment method (also known as no-till or direct drilling). Zero-Tillage minimises soil disturbance to only directly where the seed is planted^53,54^.	Specialised direct seeding machines
*Contour Farming*	Planting crops along natural land contours to minimise soil erosion and runoff, especially on slopes (also known as contour-parallel cultivation).	Standard equipment (adapted to slope contour)
*Deep Non-Inversion Tillage*	A deep non-inversion tillage treatment for mechanically loosening soil below the usual tillage depth to address compaction or improve drainage, without disturbing the soil surface^53^.	Subsoiler
*Controlled Traffic Farming*	Operating all machinery for several years only on designated tramlines to minimise soil compaction in non-trafficked areas	~ GPS-controlled machines (e.g., seeders and sprayers) ~ Implements of the same working width
*Stubble Mulching*	Mechanical treatment to chop and distribute crop residues (e.g. stubble) to aid the breakdown of crop residues and aid weed/pest control.	~ Mulcher ~ Mower/topper
*Under-sowing of Main Crop*	Planting secondary crops under the primary crop to improve soil cover and suppress weeds^55,56^.	~ Spreader ~ Specialised or adapted standard seeding technology
*Sowing Catch Crops or Green Manures*	Planting crops within crop rotation to prevent nutrient loss, improve soil structure, and add organic matter. Seed mixtures can be designed for different purposes^55–57^.	~ Specialised or adapted standard seeding technology ~ Could be performed e.g. alongside subsoiling
*Application of Biochar*	Adding charcoal-like material to soil to improve water retention, nutrient holding capacity, and carbon sequestration.	~ Manure spreaders with adapted spreading attachments ~ Specialised spreading technology
*Application of Compost*	Enriching soil with rotted organic material produced from plant material and to boost fertility^55^.	Manure spreaders with adapted spreading attachments
*Soil Condition Tests*	Performing simple tests, like spade sampling, to check if the soil is firm and dry enough to avoid damage before driven on^57^.	Spade sampling tools

#### Milling Wheat Production (Wheat dataset columns B-M)

Production standard, hectares of milling wheat grown, yield milling wheat, yield milling wheat over last five seasons, quantity synthetic fertiliser, quantity organic fertiliser, sowing density, number of biostimulant treatments, number of herbicide treatments, number of fungicide treatments, number of insecticide treatments and number of plant growth regulator treatments.

#### Structural Farm Characteristics (Primary dataset columns CD-CP)

Family members employed, farm focus (arable, livestock, permanent crop, others), full time or part-time farm, percentage of rented land, whether the soil has been assessed and a soil management plan exists.

#### Training and Advice (Primary dataset columns CQ-CZ)

Advice agricultural adviser, advice agricultural retailer, advice cantonal or national institution, consult other farmers, consult social media channels, consult publications or webpages, participation equipment demonstration, participation farmer discussion or training group, participation farm demonstration, participation course.

#### Behavioural and Attitudinal Factors (Primary dataset columns DA-EK)

Respondents’ self-assessment of their perceived influence of the weather on crop production and ambitiousness of self-set production goals.

Respondents’ self-assessment of their willingness to take risks in the domains of; agricultural production, investment in agricultural technology and crop protection.

Respondents’ self-assessment of their confidence in being able to; find solutions to arable production challenges and achieve production goals by harvest end.

The respondents self-reported importance of the following aspects in decision making;

Maximising yields, minimising input costs, minimising time or labour requirements, minimising production risks, minimising farm exposure to weeds or pests or diseases, adapting to weather patterns, adapting to farmland conditions, improving soil health or structure or fertility, improving biodiversity, minimising environmental impact, expanding farm land, adapting to crop market developments, adapting to changes in direct payment rates or regulations, seeking professional agronomic, seeking casual advice from friends or colleagues and seeking peer approval.

### Ethical approval and pre-registration

The survey campaign and research design were both approved separately by the ETH Zürich Ethics Commission as proposal 2023-N-212 as well as the FiBL Ethics Committee as proposal FSS-2023-006. Copies of the approval letters are included in the supplementary material^1^. Before launching our survey, we also submitted two research plans for pre-registration of hypotheses via the online platform AsPredicted operated by the University of Pennsylvania (link: AsPredicted). For further information on these, see AsPredicted #153145 and AsPredicted #153146 that were registered on 29^th^ November 2023.

### Survey implementation

The survey was implemented as an online survey formulated with Lime Survey and distributed via email. All eligible farms received an individualised email addressed personally to the recipient and a survey link, connected with a unique token to enable us to link the farmer responses with secondary data available for each farm. The participants were asked to give their permission for this by approving the terms and conditions we made available to them regarding how their data would be handled. By agreeing to the disclosure agreement, the farmers gave their permission for the anonymised data, that they subsequently provide through the survey, to be used exclusively for science and research purposes. Farmers were also given the option to opt out of the survey at any time, with no explanation needed. To incentivise participation in the survey we offered the opportunity to enrol in a lottery of 100 supermarket vouchers worth CHF 150 each and the option to receive a personalised results report comparing the farmers’ answers to the answers of other similar farms. The individualised reports were administered via a bespoke app created using R-Shiny (see technical validation section below for further details).

Prior to the full survey launch, a pilot survey was conducted on a random sample of 1% of eligible farms (150 farms) to test the survey’s functionality and to refine any issues. The pilot survey launched on 30^th^ November 2023, and the full survey went live six days later, on 6^th^ December 2023. The survey was closed on 31^st^ January 2024, after a response period of nearly two months.

### Data cleaning

To minimise errors already at the point of data entry, the survey was designed to allow only predefined values or plausible numeric ranges for most variables. Wherever this was not technically feasible, such as in open-text fields or free numeric input, we conducted systematic data cleaning after data collection. Data cleaning involved addressing inconsistencies and missing values. In cases where values were deemed implausible or outliers, they were either removed or corrected if sufficient data from other columns was available. This cleaning procedure was applied to variables related to plant protection product treatments, yield, sowing density, labour input, and demographic information. We include the following to illustrate the approach we took as an example (note all processing codes are available in the supplementary material which outline these decisions on a line-by-line basis):

If in the labour units column, an entry was listed as 48, which was inconsistent with the farm area, this value was corrected to 4.8 using a related column for recalculation. Similarly, we proceeded for the variable *age*: if a data entry was obviously wrong, such as a year of birth recorded as 60 instead of the demanded format YYYY (1960), and the farmer had entered the column of farming experience 40 years, the value was corrected to ‘1960’ based on logical inference. If no reliable correction could be made, the value was marked as ‘NA’ (Not Available).

To ensure anonymity, apart from removing precise geographical information we also grouped continuous variables such as age and farming experience into categories (e.g. age_group and years_experience_group). The data was anonymised, and no specific details were included that could link individual responses to specific farms. No randomisation was applied to the data. With regard to the secondary data, we also took measures to prevent identification by rounding the variables to the nearest integer (the codes for the processing of this data are also available in the supplementary material).

## Data Records

The data consists of one main dataset and two supplementary datasets^1^. Each dataset has columns representing variables (see the codebook for an elaboration of these variables), with each row corresponding to an individual farm. The main survey dataset “Primary_Data” contains 2,728 rows and 141 columns, providing comprehensive information on demographic details (B-H / CD-CP), participation in soil health programmes (I-S), management practice adoption and knowledge (T-CA), structural farm characteristics (CD-CP), training and advice (CQ-CZ), behavioural and attitudinal factors (DA-EK). The supplementary datasets can be linked to the main dataset via a common key variable (“survey_id” - column A in excel), which assigns a unique identifier to each participating farm and is consistently present across all three datasets.

All data and codes are available for download at: 10.5281/zenodo.15488101.

The supplementary dataset “Wheat_Data” includes the same farms as the main dataset, but with supplementary data for only those that grew milling wheat in the 2022/2023 farming season. Farms present in the first dataset but not in the second did not produce milling wheat during that period. It consists of 2,217 rows and 13 columns and includes data on wheat production (B-H) and plant protection treatments (I-M).

The additional supplementary dataset “Linked_Secondary_Data” includes any spatial and meteorological data that we did not collect via our survey but were able to link to the farm via the farm ID number. This data consists of variables such as rainfall totals (AK-AV), average temperatures (X-AJ), soil textural variables (T-X), production areas (E-S) and any other relevant geographical information (B-D). All sources used for this data are listed in the codebook. As per the primary data listed above, this data is also adjusted to maintain the anonymity of the farmers. It consists of 2,728 rows and 48 columns and is also available within the provided data repository.

Three accompanying PDF files (“Primary_Data_Codebook”, “Wheat_Data_Codebook” and “Linked_Secondary_Data_Codebook”) provide a comprehensive description of all variables, linking them to the relevant survey questions and offering additional explanations where necessary. Additionally, a PDF document of the complete survey questionnaire used to collect the data is included, available in English, German and French. The questionnaire outlines all survey items and response options. The R scripts in the repository include a quality check script to validate data consistency. However, due to anonymity concerns, exact farm locations are not provided. As a result, scripts that replicate visualisations, such as spatial distributions of farms are not included.

### A note on linked secondary data

We used the geodienste.ch website to extract secondary data on agricultural land use in Switzerland. The publicly available data sources, which are regularly updated by the responsible authorities for planning and monitoring purposes, provide detailed information on land use by farm. We extracted the relevant datasets for 2022/2023 and include summarised versions of this to complement our primary data collection, presented above. The secondary data from geodienste.ch is helpful for contextualising our results and enables an enriched analysis of agricultural practices in different regions via the inclusion of highly detailed and spatially explicit factors unique to each farm, such as soil texture, geomorphology and weather records. If you wish to use further geographical data - beyond the summarised versions that we include - available via geodienste.ch or any other open data provider in conjunction with the presented data, please follow the individual licensing agreements of these providers.

## Technical Validation

### Survey design verification

Throughout the execution of the data collection process, we followed a rigorous protocol and sought a wide range of advice from different people and sources over the course of 2023. We did this to ensure the technical validity of our questions and that the scope of our survey was both appropriate and in line with other contemporary surveys undertaken in Switzerland. Summary statistics comparing our survey with responses to the previously conducted surveys are presented below in Table 3.

We iteratively tested the survey with four soil scientists, three agricultural advisors and two farmers during the design of the questions and structure to get their feedback and ensure the highest possible levels of comprehensibility and functionality. Additionally, we tested the different language drafts with six native speakers (three German-speaking and three French-Speaking) to ensure that the contents were understandable, the wording was uniform and the questions identical across language versions.

### Survey responses verification

#### Response rate

In total, the survey was sent to 15,023 eligible farms, of which 2,728 fully completed the survey, yielding a completion rate of 18.16%. This response rate is comparable with, or higher than, similar agricultural surveys that have been conducted in Switzerland^45–49^. From the 3,515 farmers that started the survey 779 farmers started but did not finish the survey. Eight responses were excluded due to incomplete or invalid data, specifically, if respondents indicated they were not involved in any farm management decisions. The click-through-to-completion rate for the survey was 77.24%, which is also comparable to other studies in Switzerland (3,515 started vs 2,728 finished).

**Fig. 3:**
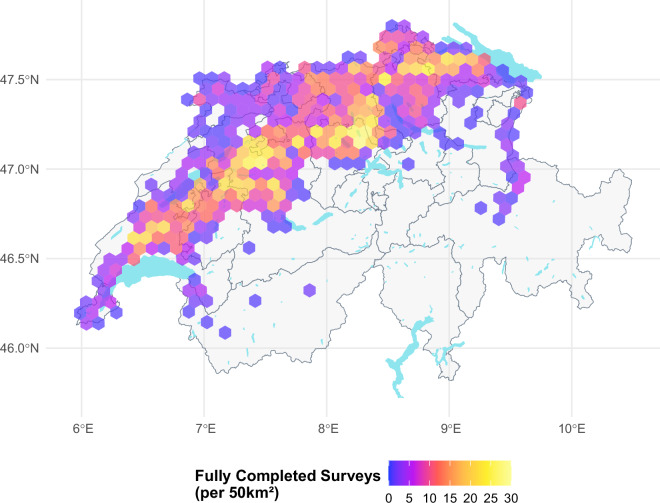
This figure illustrates the geographical distribution of all farms that both received a survey invitation and fully completed the survey. The majority of farms are concentrated in valley zones, where arable farming is most viable due to favourable environmental conditions. The grey lines outline the cantons, providing a spatial reference for orientation.

**Fig. 4:**
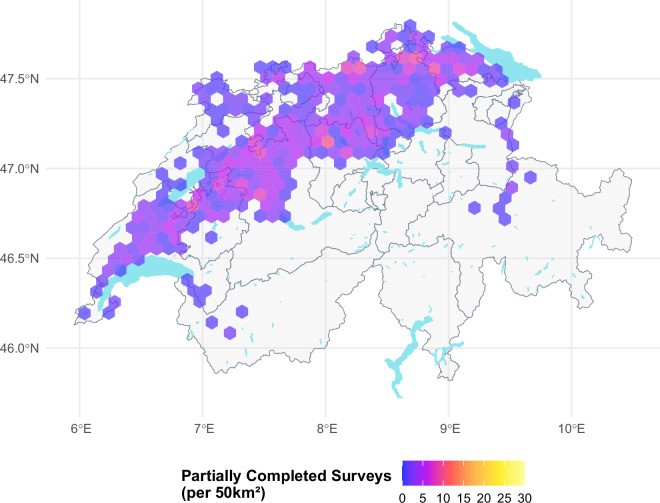
This figure illustrates the geographical distribution of all farms that initiated the survey but did not reach completion. These partial responses are similarly clustered within the valley zones. The grey lines outline the cantons, providing a spatial reference for orientation.

**Fig. 5:**
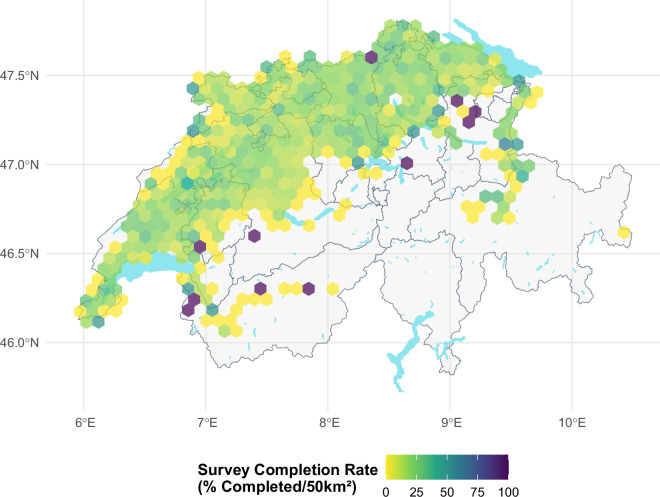
This figure displays the percentage of farms that completed the survey out of all contacted farms that were eligible for the survey in each 50 km^2^ hexagonal grid cell. The values represent relative proportions of completed surveys within each hexagonal cell, rather than absolute counts. Completion rates in cells with very small sample sizes may be statistically less reliable. The grey lines outline the cantons, providing a spatial reference for orientation.

The farms who responded to our survey span the diverse agricultural landscapes of Switzerland and the two major cultural regions (French and German-speaking Switzerland). To verify the representativeness of the survey responses, we compared the geographical distribution of farms that completed the survey (Fig. 3), those that started but did not complete (Fig. 4), and the overall completion rate (Fig. 5). There is no indication of systematic no-response bias across the regions and higher farm numbers within a cell are often linked to the higher number of farms present within the area. It should be noted that the Italian-speaking part of Switzerland is not included in this study due to its distinct agricultural practices and environmental conditions, which differ significantly from those in the other regions north of the main alpine chain, as well as the limited number of participants who met the survey’s requirements. It should be noted that we can only present the geographic distribution and structural summary statistics of the response trends for the farms that were possible to link to the secondary data. This explains the slight discrepancy between the number of observations in our cleaned datasets and the structural comparisons presented below in Tables 2 and 3.

**Table 2:** Mean and (standard deviation) are reported for continuous variables, whilst the percentage of respondents in a category are reported for categorical variables.

Variable	Target Population ☨	No Answer Farms	Partial Answer Farms	Full Answer Farms
Utilised Agricultural Area (ha)	32.95 (23.39)	32.48 (23.58)	32.95 (23.04)	34.95 (22.56)
Arable Area (ha)	21.18 (17.05)	20.71 (17.25)	21.08 (16.09)	23.17 (16.29)
Milling Wheat Area (ha)	4.27 (5.38)	4.17 (5.47)	4.25 (4.69)	4.71 (5.15)
Proportion of Arable Area Under Temporary Grassland (%)	29.66 (24.37)	30.66 (24.70)	28.38 (23.95)	25.81 (22.63)
Valley Zone (% Yes)	75.17	74.13	75.8	79.34
Number of Farms (No.)	15,013 *	11,498	779	2,728

A table of descriptive statistics from the here presented dataset versus the total population of farms that met our selection criterion. We include means and standard deviations of the whole survey population versus three separate categories of farm responses to the survey a) no response, b) partial response and c) full response - i.e. the farms that are included in the datasets presented. *

*Note that we were not able to locate official federal data covering the farming population in Switzerland that was split by farms growing crops on arable land. Therefore, we extracted structural summary statistics for all farms that we contacted using publicly available data from geodienste.ch. We believe that this approach provides a more appropriate means of testing whether there are any systematic sampling biases than comparing data for the entire Swiss farm population. We can only present the geographic distribution and structural summary statistics of the response trends for the farms that were possible to link to the secondary data. This explains the slightly different number of observations between our cleaned datasets and the figures presented in this table (15,013 versus 15,023).

☨ Note that here the target population refers to the entire population of Swiss farms which met our outlined selection criteria of: a) the farm grew wheat in the preceding season (2021/2022), and b) the farm managed at least 3 hectares of arable land in the preceding season (2021/2022) and c) that arable land must have comprised at least 20% of the total farmed area in the preceding season (2021/2022).

**Table 3:** Mean and (standard deviation) are reported for continuous variables, whilst the percentage of respondents in a category are reported for categorical variables.

Variable	Rees *et al*.^1^ (Data Presented)	Möhring *et al*.^49^	Späti *et al*.^48^	Kreft *et al*.^50^
Share Rented (%)	37.50 (28.67)	34.94 (28.87)		
Labour Units (full time equivalents)	2.36 (4.40)	1.68 (1.18)	1.86 (2.14)	
Manager Age (years)	47.54 (10.28)	47.08 (9.37)	46.50 (10.37)	49.03 (10.13)
Female (% Yes)	5.06			6.9
French Speaking (% Yes)	22.62	21.9		24.34
Valley Zone (% Yes)	79.18		76.56	
Organic (% Yes)	16.14*		12.92	25.35
Integrated Production (% Yes)	47.90*	100		40.65*
Reduced Tillage Scheme Enrollment (% Yes)	58.14	59.19 ^☨^		47.68
Final Number of Usable Responses (No.)	2,728	1,105	418	797
Response Rate (Usable % of asked)	18.16	23.27	8.74	

A table of descriptive statistics from the here presented dataset versus the three contemporary survey datasets that focused on arable farming in Switzerland. Namely those of Möhring *et al*. (2022), conducted in 2019, Späti *et al*.^48^ conducted in 2021 and Kreft *et al*.^50^, conducted in 2024. These descriptive statistics are also shown alongside general statistics of the entire agricultural sector - including all farm types - of Switzerland from the Federal Office of Statistics for the year 2023.

* Calculated on the basis of the subset of farms producing milling wheat due to the fact that integrated production farms register per crop and not for the whole farm - i.e. they may produce some crops conventionally and others under the integrated production standard.

^☨^ Note that the schemes were not exactly the same in 2019 when the data was collected by Möhring *et al*. (2022) compared to when we collected our data in 2023/24. The soil cover scheme was not recorded, and the reduced tillage scheme was split into two parts - direct seeding and mulch seeding. We report the percentage of farms from Möhring *et al*. (2022) who were enrolled in mulch seeding and/or direct drilling.

### Structural comparisons of respondents

To further assess the representativeness of our sample and understand whether any systematic biases and/or respondent self-selection biases are present, we compare descriptive farm structural statistics of all farms that were contacted during our survey campaign by their overall response state.

Overall, the sample of respondents that completed the survey are very comparable to the entire population, as well as the two cohorts of non-complete respondents. However, there are some noticeable differences, firstly in terms of land usage and farm size (Table 2). For instance, the farms that completed the survey tended to farm slightly larger areas and also had a lower proportion of temporary grassland in the rotation. Temporary grassland share acts as a proxy for the degree of specialisation in livestock production, and higher temporary grassland shares are typically found in the mountain and hill regions. This suggests that farms - of the farms that met the selection criteria - which are relatively more engaged in livestock production are slightly less represented in the survey. Additionally, as can be seen in Fig. 5 and Table 2, farms in the border regions, which are geographically consistent with the areas where arable farming becomes more constrained by geography (e.g. in the mountain and hill zones shown in Fig. 1), also had a lower tendency to respond to the survey.

### Comparison with other surveys

#### Farm and management characteristics

As a further descriptive validation of the dataset collected, we now present a comparison of farm management and farm structural statistics within Table 3. We include the three most recent surveys undertaken at a large scale across Switzerland that focused on wheat production and arable farming - or in the case of Späti *et al*.^48^ across multiple cantons^48–50^. We include the variables that were covered in our survey - which took inspiration from the survey campaigns here presented - to indicate the representativeness of our sample and the validity of our dataset. In general, there is very good agreement between the data collected in these three surveys and the data we collected that is presented within this data descriptor.

#### Milling wheat production

We also include a comparison table (Table 4) to validate the responses relating to milling wheat production, which is available for the subset of farms that produced milling wheat in 2022/2023. This data is named “Wheat_Data” within the data repository. Whilst the official data available from the Swiss national agricultural research institute (Agroscope) comes from a sample size smaller than the sample of respondents in the dataset presented in this descriptor, there is good agreement between sources in terms of average yields^51^. Additionally, the relative level of crop input usage of our sample of farms are within the recommended - and legally allowed - application levels for each production system.

**Table 4:** Mean and (standard deviation) are reported for continuous variables.

Variable	Data Presented (Rees *et al*. 2025)			Official Statistics		
Production System	Conventional	Integrated Production	Organic	Conventional ‡	Integrated Production ‡	Organic ‡
Wheat Yield (tonnes/ha)	6.70 (1.16)	6.10 (0.93)	4.52 (1.08)	6.62	5.92	4.27
Synthetic Fertiliser (kg N/ha) *	106.58 (38.59)	97.56 (37.58)	0 (0)			
Organic Fertiliser (kg N/ha) *	32.47 (30.90)	36.12 (32.74)	72.73 (38.10)			
Sowing Density (kg seed/ha)	178.76 (25.22)	180.23 (25.03)	196.98 (24.48)	160–200 ^L^	160–200 ^L^	180–220 _L_
Synthetic Crop Protection Expenditure (CHF/ha) ^☨^	141.54 (83.73)	65.71 (54.11) ^☨^	0 (0)			
Milling Wheat Area Grown (ha)	6.40 (5.64)	6.07 (5.35)	4.46 (4.02)	4.53	4.23	3.25
Milling Wheat Arable Area Share (%)	25.72 (10.40)	24.39 (10.30)	21.42 (9.28)	20.51	19.78	19.75
Number of Observations (N)	660	948	374	132	189	59

A table of descriptive statistics for milling wheat production per production system in Switzerland. We present this alongside the data reported by Agroscope for the central evaluation of farm performance in Switzerland in the year 2023. Within the category that we label as organic, we also include the farms that produce biodynamically under the Demeter label as data that splits organic farms into organic and biodynamic production is not available.

* Note that the reference amount of Nitrogen application from all sources together for winter milling wheat with a target yield of 6 tonnes per hectare is 140 kg N/ha. If targeting a yield of 8 tonnes/ha the application may be increased by up to 20 additional kg N/ha. More specific additions and deductions are allowed on the basis of factors such as soil type and previous crop^33^.

^☨^In general, farmers producing milling wheat under the integrated production system are allowed to apply herbicides - unless they take part in the pesticide free scheme - but are prohibited from applying fungicides, insecticides and plant growth regulators. We only included herbicides, fungicides and plant growth regulators in our calculation of crop protection expenditure using the reference costs of CHF 71, CHF 97 and CHF 56 / single treatment respectively - as used in the Agridea Deckungsbeiträge booklet 2023^58^.

^‡^The official statistics on milling wheat production are extracted from the Agroscope Betriebszweigergebnisse 2023. For making their calculations for 2023 they used a sample of 132 conventional wheat producers, 189 integrated production wheat producers and 59 organic wheat producers. Standard deviations of the estimates are not provided by Agroscope^51^.

^L^Recommended application rates from the practical handbook used by farmers in Switzerland^33^.

### Feedback and results dissemination

Following the completion of the data collection phase we took further steps to get feedback on the quality of the responses that we received from checking the data with colleagues to sharing the results with the farmers and allowing them to give us feedback. One of the incentives for encouraging farmers to participate in our survey was the offer of receiving a personalised benchmarking report. Within this, the performance of the farm in terms of the number and extent of soil management practices used were directly compared to other similar farms. Such results reports have also been offered in other contemporary surveys in Switzerland^46,47^.

Because of the very large number of responses, we opted to do this in a digital format via the creation of a bespoke R-Shiny app that we sent to the 2,001 farmers who requested the benchmarking report (the application is free to view at: https://fibl.shinyapps.io/InBestSoil/). Each farmer was issued with a unique 15-character token which enabled them to benchmark the answers they gave in the survey against other farms. The application is interactive in that farmers could filter by many different factors – i.e. farm size, geographic area and production system – so that they could compare the results in whatever way they liked.

By developing the benchmarking application, we sought to give farmers actionable insights derived from their own data, enabling them to assess their practices in relation to similar farms and identify potential areas for improvement. Development of this benchmarking application began in March 2024, after the end of the survey campaign on the 31^st^ of January 2024. The above linked version of the live application was sent via email to the farmers on 11^th^ of June 2024. The farmers were able to provide feedback on the survey and application. While the overall feedback response rate was low, the feedback received was highly positive. The benchmarking platform was accessed for approximately 400 hours between launch on the 11^th^ of June 2024 and mid-August 2024, relating to an average usage of approximately 15 minutes per responder who was given access. Aside from sharing the application with the farmers who participated, we also presented the application to the Swiss Federal Office of Agriculture on 22^nd^ October 2024 and received very positive feedback on the survey, data collected, and the scope of the benchmarking application created.

## Usage Notes

The datasets that we provide via Zenodo, and described via this data descriptor, can be used for analysing the adoption dynamics of environmentally beneficial arable soil management practices within Switzerland. This can be insightful for informing policymakers, as well as agricultural extension organisations, with regard to how to better integrate and expand the uptake of these practices by farmers. This dataset also allows a thorough comparison of the differences in adoption rates between three separate production systems. To this end, the datasets can also be used to identify drivers and barriers to adoption (at the extensive and at the intensive margins), understand farmer decision-making behaviour, joint adoption decisions of complementary practices vs. crowding out effects, perceived challenges and attitudes towards given production goals within, and between these three production systems. Beyond ex-post analyses, these behavioural components can also be employed for the parameterisation of farm-level models.

The primary dataset is complemented by detailed milling wheat production data covering a large proportion of the participating farms. These data may be used to analyse productivity and wheat enterprise performance whilst also taking into account highly detailed, and often omitted, geographic and environmental constraints - as supplied via the linked secondary data. To the same end, this data can also be utilised to inform ex-ante modelling efforts.

We recommend that any studies looking to utilise this dataset for an analysis should further screen the data for outliers using a multivariate outlier detection algorithm such as BACON^52^. In our own subsequent research using this data we follow this same protocol.

## Data Availability

The code and the survey data are available on Zenodo in the following repository: 10.5281/zenodo.15488101.
